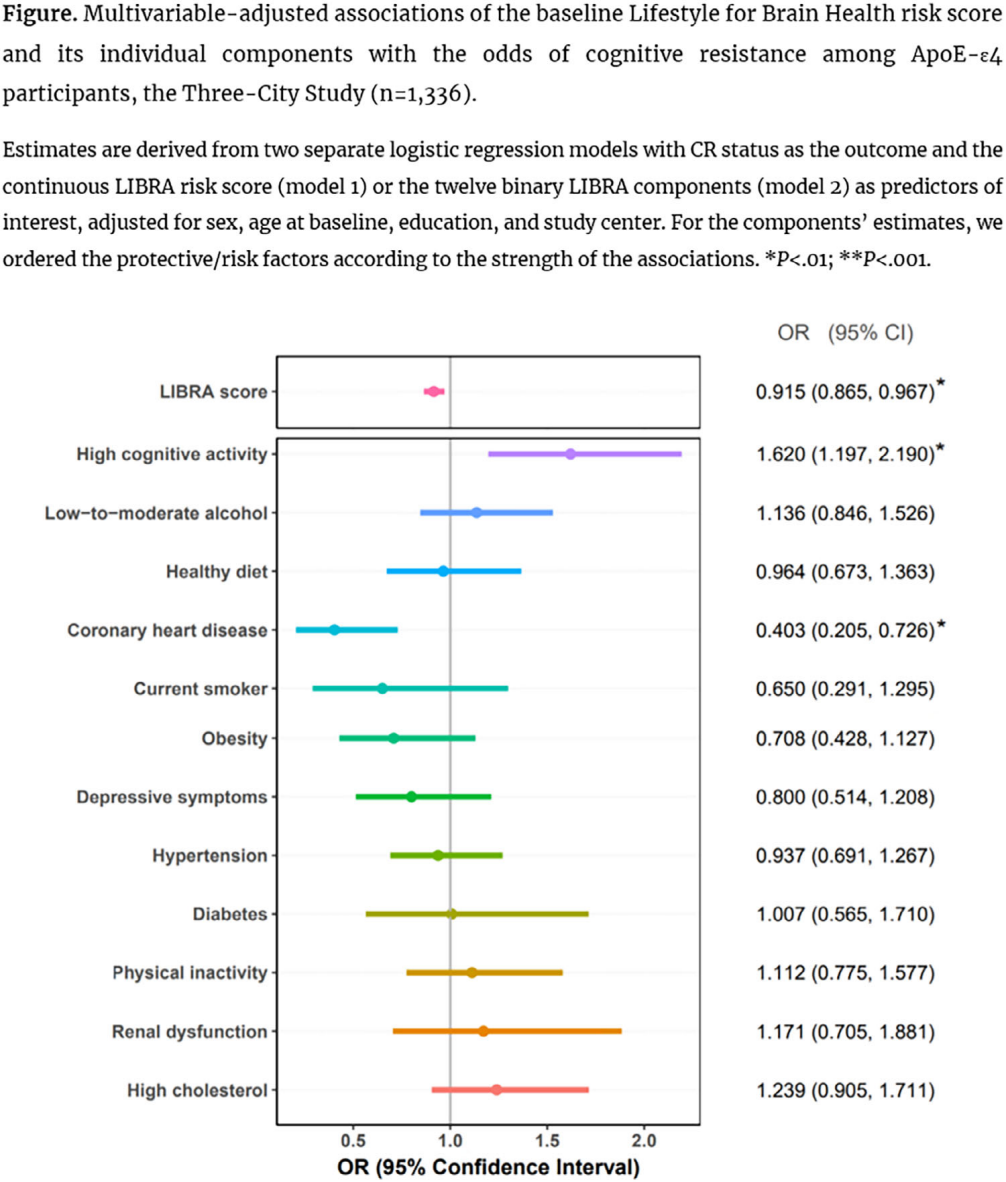# Associations of the LIBRA risk score with cognitive resistance to susceptibility genes for dementia

**DOI:** 10.1002/alz.085538

**Published:** 2025-01-09

**Authors:** Maude Wagner, Jeanne Neuffer, Quentin Le Grand, Aniket Mishra, Claudine Berr, Christophe Tzourio, Stéphanie Debette, Catherine Helmer, Cécile Proust‐Lima, Cécilia Samieri

**Affiliations:** ^1^ Rush Alzheimer’s Disease Center, Rush University Medical Center, Chicago, IL USA; ^2^ Bordeaux Population Health Research Center, Inserm U1219, University of Bordeaux, Bordeaux France; ^3^ INM, Univ Montpellier, INSERM, Montpellier France

## Abstract

**Background:**

Some older adults succeed in maintaining excellent cognition despite high genetic risk for Alzheimer’s disease (AD), reflecting cognitive resistance (CR) to potential neuropathologies. Although the etiological factors for CR are still unknown, some literature suggests that environmental/lifestyle risk factors may contribute to offset, or at least reduce, the effect of AD‐related genes on cognitive decline and dementia risk. Yet, how modifiable lifestyle and health related risk factors may promote CR in genetically at‐risk individuals remains to be elucidated.

**Method:**

We selected 6,774 dementia‐free participants from the Three‐City study cohort (≥65 years), who underwent ApoE and genome‐wide genotyping, at least 2 neuropsychological evaluations over time, and had information for the LIfestyle for BRAin health (LIBRA) score at baseline. LIBRA is a validated weighted score of 12 modifiable risk factors, including unhealthy lifestyle, poor cardiometabolic health, depression and renal dysfunction, with higher scores denoting increased lifestyle‐related dementia risk. We defined high genetic risk through ApoE‐ε4 status alone or combined with high AD‐specific genetic risk scores (GRS) beyond ApoE. We operationalized the concept of CR to genetic risk based on the individual slopes of global cognition estimated using latent process mixed models adjusted for demographics and genetic risk. CR status was assigned to genetically at‐risk participants with an adjusted cognitive slope in the slowest 25% of the overall population. Finally, focusing on genetically at‐risk individuals, we examined the associations between the LIBRA score, its individual components, and CR status using separate logistic regression models adjusted for sex, age, education, and study center.

**Result:**

Among genetically at‐risk participants (n = 1,336 ε4‐carriers; n = 2,065 ε4‐carriers/GRS‐high), 237 (18%) ε4‐carriers and 470 (23%) ε4‐carriers/GRS‐high were cognitively resistant to susceptibility genes. Each unit increment in the LIBRA risk score at baseline was strongly associated with lower odds of CR in ε4‐carriers (odds ratio [OR] = 0.915; 95% confidence interval [CI] = 0.865‐0.967; *P* = .002; **Figure**) and ε4‐carriers/GRS‐high (OR = 0.883; 95%CI = 0.847‐0.920; *P*<.0001). When examining LIBRA components, cognitive activities and history of coronary heart diseases showed the strongest independent associations with CR (all P<0.01; **Figure**).

**Conclusion:**

Genetically at‐risk older adults can develop cognitive resistance, which may be substantially promoted by prevention programs targeting lifestyle modifications and health management.